# Dystocia Due to Male Type of Pelvis and Hydrocephalus

**Published:** 1889-03

**Authors:** Eugene A. Smith

**Affiliations:** 66 High Street; Buffalo, N. Y.


### *DYSTOCIA DUE TO MALE TYPE OF PELVIS AND HYDROCEPHALUS.*

BY EUGENE A. SMITH, M. D., Buffalo, N. Y.

On the evening of December 10, 1888, I was called to attend Alice ———, a primipara, aged thirty-five, who had been having labor pains for twenty-four hours. She was a frail, undersized woman, lacking in intelligence, and having a congenital deformity of both auricles, with deafness.

An examination revealed a pelvis of the male type; the acute pubic angle and the rigidity of the soft parts making the entrance of the examining finger extremely painful. The cervix was thick and rigid, the os uteri dilated to the size of a ten-cent piece. The waters had been dribbling away some time, the attending midwife being unable to state just how long. Patient's pulse was 120; uterine pains were severe, but brief.

Owing to the pain caused by an examination, and the undilated cervix, I failed to make out the presentation. Abdominal examination revealed a large tumor, but the bony feel of the head was not to be found, and, as the fetal heart was faint, but distinct, in the left inguinal region, I considered the head to present L. O. A., and to be obscured by liquor amnii and its low position.

Hot carbolized douches and syrup of Dover's powder were ordered for their relaxing effect on the rigid cervix. Returning to the case four hours later, the cervix was only dilated to the size of a quarter dollar. Three ten-grain doses of chloral were given at half-hour intervals with a marked relaxing effect on the cervix. Half an hour after the last dose, it was fully dilated. But the presenting part still remained at the pelvic brim. As the patient's pulse was weaker and her pains were no stronger, but rather more spasmodic, I now sent for Dr. J. S. Smith.

Under chloroform, an axis-traction forceps (Neal's modification of Tarnier's) was applied to the presenting part, which was now found to be the breach, R. D. A. The forceps slipped once, owing to the patient, who was not fully anesthetized, jerking away from the operator. The breach was readily brought down on another trial, and the legs used as traction factors. But though in haste to deliver the body, Dr. Smith could not even bring the arms down. I relieved him, and after some effort succeeded in sweeping the arms over the front of the chest, and bringing them out. Pulsation in the cord had ceased.

Half an hour was then spent in trying to bring the head down, without avail, except that the diagnosis of hydrocephalic fetus was made from the size of the uterine tumor above the pelvic brim, and the presumptive evidence furnished by the fact that the fetus had a lumbar spina bifida, and also double talipes varus. Dr. Smith now attempted to use a perforator, but, owing to the small pelvic cavity, could only reach the head, after some effort, by passing the instrument under the pubis, through the nape of the neck, and pushing it up the spinal column, and through the foramen magnum. A steady stream of water at once gushed out, and a few moments later the fetus, with a totally collapsed head, fell from the mother's parts, closely followed by the placenta, the cord not broken.

The uterus contracted firmly and stayed so. No laceration occurred beyond a small tear in the posterior vaginal walls, perhaps due to the slipping forceps. A corrosive sublimate, 1 4,000 douche, was given and carried into the uterus. The patient was ordered anodynes and stimulants.

During the operative work, the patient's pulse mounted and became weaker. She emerged slowly from the chloroform, and failed to rally from her state of depression. Twelve hours after labor her pulse was 160; temperature, 102°. She seemed bright, but heart failure was steady until twenty-four hours after delivery, when she died.

The fetal head, when distended with water, measured seventeen and three-quarter inches from the chin over the vertex to the occiput; nineteen inches measuring under the chin and over the head from ear to ear; and twenty inches measuring the hat-crown circumference. A man's hat, size seven and one-eighth, would fit the head. The child was in other respects fully, though abnormally, developed, the mother stating that she had gone a month overtime.

66 HIGH STREET.

